# Improving the Accuracy of Decawave’s UWB MDEK1001 Location System by Gaining Access to Multiple Ranges

**DOI:** 10.3390/s21051787

**Published:** 2021-03-04

**Authors:** Antonio R. Jiménez, Fernando Seco

**Affiliations:** Centre for Automation and Robotics (CAR), Consejo Superior de Investigaciones Científicas (CSIC)-UPM, Ctra. Campo Real km 0.2, La Poveda, Arganda del Rey, 28500 Madrid, Spain; fernando.seco@csic.es

**Keywords:** indoor localization, ultrawideband radio (UWB) based positioning, accuracy, robust positioning methods, wireless sensor networks, bluetooth low energy (BLE)

## Abstract

The location of people, robots, and Internet-of-Things (IoT) devices has become increasingly important. Among the available location technologies, solutions based on ultrawideband (UWB) radio are having much success due to their accuracy, which is ideally at a centimeter level. However, this accuracy is degraded in most common indoor environments due to the presence of obstacles which block or reflect the radio signals used for ranging. One way to circumvent this difficulty is through robust estimation algorithms based on measurement redundancy, permitting to minimize the effect of significantly erroneous ranges (outliers). This need for redundancy often conflicts with hardware restraints put up by the location system’s designers. In this work, we present a procedure to increase the redundancy of UWB systems and demonstrate it with the help of a commercial system made by Decawave. This system is particularly easy to deploy, by configuring a network of beacons (anchors) and devices (tags) to be located; however, its architecture presents a major disadvantage as each tag to be located can only measure ranges to a maximum of four anchors. This limitation is embedded in the Positioning and Networking Stack (PANS) protocol designed by Decawave, and therefore is not easy to bypass without a total redesign of the firmware. In this paper, we analyze the strategies that we have been able to identify in order to provide this equipment with multiple range measurements, and thus enable each tag to be positioned with more than four measured ranges. We will see the advantages and disadvantages of each of these strategies, and finally we will adopt a solution that we implemented to be able to measure up to eight ranges for each mobile device (tag). This solution implies the duplication of the tags at the mobile user, and the creation of a double interleaved network of anchors. The range among tags and the eight beacons is obtained through an API via a wireless BLE protocol at a 10 Hz rate. A robustified Extended Kalman filter (EKF) is designed to estimate, by trilateration, the position of the pair of mobile tags, using eight ranges. Two different scenarios are used to make localization experimentation: a laboratory and an apartment. Our position estimation, which exploits redundant information and performs outlier removal, is compared with the commercial solution limited to four ranges, demonstrating the need and advantages of our multi-range approach.

## 1. Introduction

The location of devices or people is becoming increasingly important. While global navigation satellite systems (GNSS) have become quite successful in outdoor environments (with unrestricted view to the satellite constellations), there is not yet an equivalent system operative indoors. Devices designed for this purpose receive several names in the literature: Local Positioning Systems (LPS), Indoor Positioning Systems (IPS), Real Time Location Systems (RTLS), etc. [1]. Among the several technologies employed for indoor localization (ultrasound, inertial, RF-fingerprinting, light, …) [2,3], ultrawideband (UWB) [4] radio has shown great promise given their capability to measure delays between RF emission and reception with better than nanosecond accuracy, translating into expected positioning accuracy of a few centimeters [5]. At the laboratory level, a few UWB characterization papers analyze the performance of UWB radio ranging and positioning. In [6], a performance comparison among ten impulse-radio UWB localization systems is referenced. However, in real operating environments, this accuracy is affected by the presence of obstacles in the environment, such as walls, furniture, or even people. Robust positioning or navigation in spite of these circumstances is a challenging research topic [7].

Positioning systems based on range multilateration, such as UWB, only perform optimally in open environments where obstacles are absent or there are no significant RF reflections. In this line-of-sight (LOS) conditions, the measured signal arrival times correspond to the physical distance between RF emitters and receivers. The only significant problem in LOS conditions is the destructive interference with ground reflected signal causing fading [8], but is relevant at large distances (mainly outdoors). Non line-of-sight (NLOS) conditions, such as obstacles and reflectors, introduce range outliers which may change the estimated position by a few meters or even prevent from achieving a solution at all. Unfortunately, NLOS conditions prevail in indoor environments, so all location-based applications that make use of UWB technology indoors must cope with outliers and propagation models.

Additionally, other NLOS conditions appear when the human body attenuates the UWB signal transmitted from tag to anchor, causing errors larger than 1 m as reported in [9]. They propose an original method that requires a human-body RF shadowing model and the estimation of the relative heading of the moving person, assuming a given attachment of a tag on the person to locate.

Robust outlier detection algorithms have been developed and used especially in the related fields of ultrasonic positioning and global navigation satellite systems (such as the GPS), where they are often studied as integrity monitoring techniques [10]. Counting with enough measurement redundancy, these methods permit estimating position with only the physically meaningful ranges. For navigation or tracking of mobile targets, filtering methods such as Bayesian filters are commonly used. For maximum flexibility, these methods can be adapted with robust range error statistics which include both true ranges and outliers [11]. In theory, use of proper statistics for the range error permits obtaining maximum likelihood (ML) position estimates [12].

When non redundancy is available, due to working only with the minimum number of ranges for trilateration, then temporal filtering, such as median filters or moving averaging are employed, as in this [13] UWB localization implementation. This kind of temporal in-range median-based solutions can circumvent the presence of sporadic outliers but fail when those errors are systematic. Other approaches that try to cancel outliers on the individual ranges, before the trilateration, are based on machine learning (ML) methods, such as k-nearest neighbors, Gaussian Processes, or Neural Networks [14,15,16]. However, methods based on learning are in many occasions invalid when changing the location site or if the conditions in the space change with time.

Other approaches search for the improvement of UWB position estimates by fusing with other complementary sensors, such as inertial measurements units (IMU) or GNSS [17]. Of course, these approaches are desirable and effective, but are out of the scope of this work, where we focus on the analysis of multi-ranging redundancy for independent (UWB-only) position estimation using a specific commercial device and their limitations to gain access to multiple ranges.

Apart from sensor fusion, the filtering of individual ranges by median or sophisticated ML, as reviewed above, can definitively help to improve position estimation. However, to be able achieve a true robust positioning solution, independent from particular learnt models, it is necessary to have redundant distance measurements. This is the most effective way to rule out those ranges that are significantly erroneous (outliers), and to give more weight to the subset of ranges that operate in line of sight with a normal error distribution. Redundancy in measurements is key to providing robust estimates to outliers even in non-modeled environments [6,18,19,20].

The use of redundant estimation methods is sometimes limited by the use of commercial UWB equipment. Currently, there is a very successful commercial equipment, distributed by the company Decawave, which has many operational advantages for the deployment and use of a network of *anchor* (devices to be fixed in the environment with known positions) and a set of tags (mobile devices that we want to locate). This system seems to have found widespread use within the scientific community, as a large number of published works use it in their experimental tests. However, this architecture presents a major disadvantage from our point of view: each tag to be located can only measure ranges with a maximum of four anchors. The same Decawave system that is going to be explored in this paper (MDEK1001) was recently studied by Delamare [21] and found that using only four ranges was limiting, and they concluded that a larger number of anchors should be used for better accuracy.

In case of using a positioning system with a limitation in the number of ranges to anchors, such as MDEK1001, in general there would be no redundancy at all. This causes the impossibility of trilaterating without ambiguity whenever one of the four ranges is lost. We are assuming that four ranges are needed to locate in 3D, or at least that there is one single range in redundancy if the space of interest is physically restricted. For example, if the beacons are placed on the ceiling of a room, only the solution below them is a feasible location. This four range condition is in our opinion quite limiting for a good positioning in challenging scenarios like indoors. This ranging limitation is embedded in the Decawave’s measurement protocol itself (PANS), and therefore it can not be easily bypassed without having to redesign much of the protocol in the firmware of all Decawave devices, in a variety of different working modes (tag, anchor, listener, or gateway) [22]. The redesign of a protocol requires strong knowledge in the UWB technology which includes critical factors such as synchronization, two-way-ranging (TWR) [23], clock drift correction, or power self-calibrations [24].

In this paper, we analyze the strategies that we have been able to identify in order to provide this Decawave UWB equipment with multiple range measurements, and thus enable each tag to be positioned with more than four distances, in a redundant and robust way. We will also analyze how and where to obtain all the ranging information, needed for robust trilateration, from a Decawave network. Knowing if ranging information is distributed between nodes, or centralized, is a relevant aspect to consider when implementing the robust location solution.

We will explain in this paper the advantages and disadvantages of each of the different alternatives we discovered to gain access to multiple ranges in this Decawave UWB equipment. Finally, we will present the solution that we implemented in an inexpensive way to be able to measure up to eight ranges for each tag. This solution is implemented and tested, using several communication protocols available through the API (Application Program Interface) in each node firmware: (1) UART protocol wired by USB, (2) wireless BLE protocol. In this paper, the positioning results using Decawave’s 4-range mode, and our robustified 8-range mode, are compared in two different scenarios: (1) an indoor office environment with common obstacles such as furniture or desktop computers, and (2) in a residence apartment with common household, a much more challenging scenario. We will analyze the gain in accuracy obtained with the proposed multi-range (8-range) solution, and demonstrate how the same approach (same sensors, EKF parameters, and NLOS models) can be applied in totally different scenarios with the same rate of performance improvement, which is one of the contributions of the paper, apart from the proposal of a way to make possible the use of multiple ranges in one of the most used and successful pieces of UWB equipment in research labs and corporate solutions. The need of learning or adaptation in the algorithms for different scenarios (common in many research papers [14,15,16]) is something that we were able to avoid, which is very convenient and practical in real life applications.

The paper is structured in the following sections: Section 2 presents the measurement and communication protocol used natively by the Decawave DWM1001 equipment. In Section 3, the alternatives we have found to measure multiple ranges are shown. In Section 4, we analyze the UWB radiation pattern in pairing tags. In Section 5, we show a multi-range system deployment in a laboratory test site, the implemented EKF-based solution, and the positioning results with both approaches (the original four ranges and the proposed one using eight ranges). An additional experimentation in an apartment is presented in Section 6 in order to verify that conclusions apply for different set-ups and anchor deployments even with the same sensors, models, and algorithm parameters.

## 2. MDEK1001 Architecture, Pans Communication Protocol, and APIS

This section describes Decawave’s UWB location equipment, its working architecture, the PANS communication protocol, and its limitations, as well as the programming interfaces (APIs) to gain access to the UWB ranging information. Some information has been extracted from the manufacturer manuals [22], but other operating principles and limitations have been researched via direct communications with technical support staff and our own tests. We provide the whole conclusions here as a reference.

### 2.1. General Characteristics of the MDEK1001 Equipment and Its DWM1001DEV Modules

**Figure 1:**
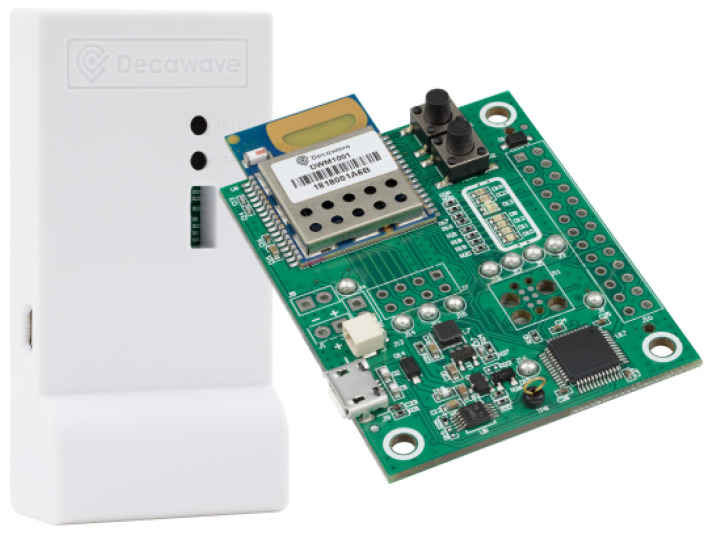
Configurable UWB module (DWM1001DEV) in the MDEK1001 evaluation kit from Decawave (the kit contains 12 such modules); left: housing; right: DWM1001 circuit board with the antenna on top [22].

Decawave’s UWB evaluation kit (MDEK1001) [22], shown in Figure 1, is described by the manufacturer as an “out-of-the-box scalable RTLS network solution.” It consists of twelve DWM1001DEV modules, each of which can be configured as an anchor or a tag; more advance modes (listener and gateway) will be explained later. Devices configured as anchors are intended to be placed in a fixed position in the environment as beacons, and the nodes configured as tag are used on the mobile device that we want to locate. A tag has the ability to measure ranges to various anchors, and locally, each tag can calculate independently its own location using the received ranges to anchors. So that each tag can locate itself, apart from ranges, they receive from the anchors their 3D coordinates; data which are initially calibrated and stored in each anchor. Therefore, the location is private to the tag, and the anchors do not register ranges. Only tags do.

**Figure 2:**
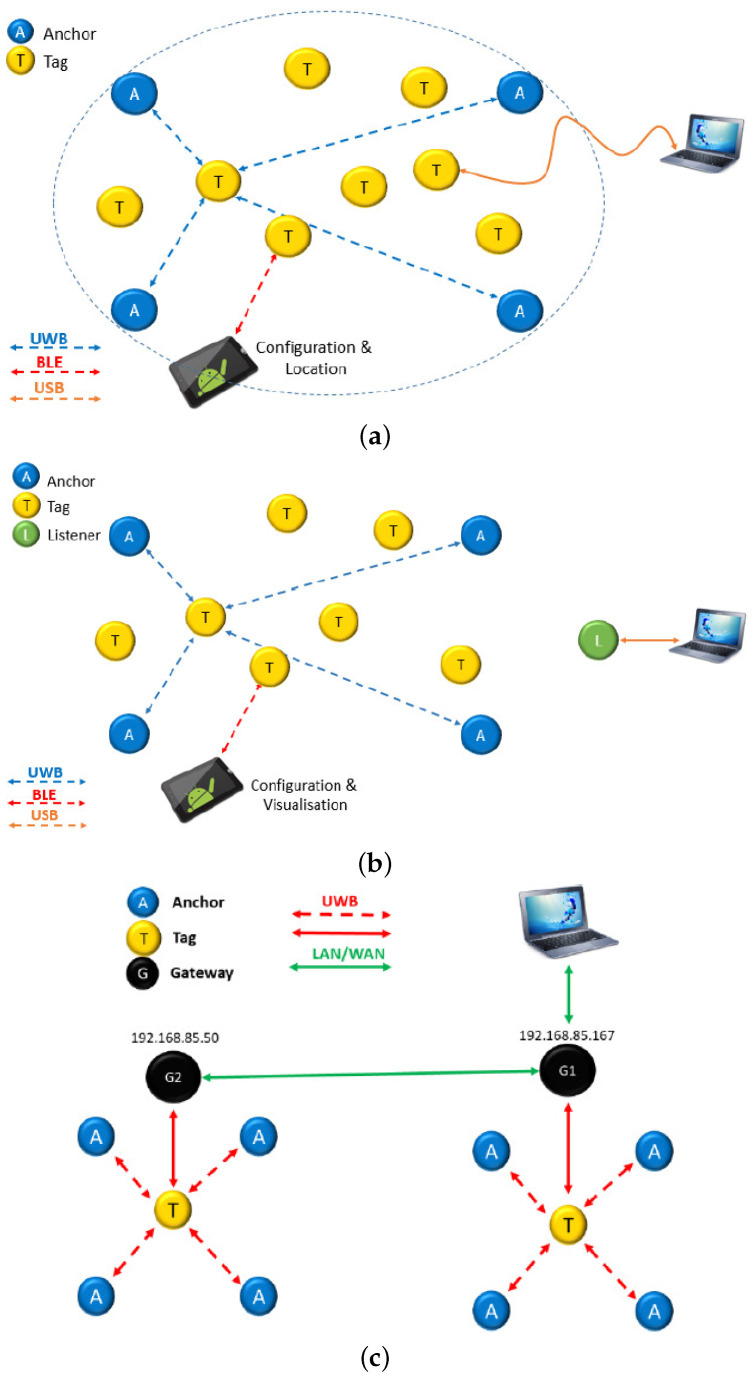
Different configurations of the DWM1001DEV modules to create a location network, and ways of monitoring: (**a**) normal mode with connection to individual tags (access to position of that tag and four ranges); (**b**) extended mode with listener to access positions of all tags in the network (not ranges); (**c**) extended mode with gateways to increase scalability of the deployment and also get access to positions of all the tags in the network (not ranges) [22].

The typical configuration for using the localization system is shown in Figure 2a, where there are eight tags and four anchors, and each tag measures ranges to a maximum of four anchors at a time (this is a limitation of the PANS protocol as we will see later). The only way to extract the ranges and positions of each tag is to get connected individually, by USB or BLE to a PC/Tablet, to each of the existing tags.

A set of tags and anchors must be associated with the same *network* (cluster) in order to be able to obtain location information for all of them. One of the anchors in the network must be configured as a *master*, which is the one that keeps the timing and synchronization of all modules in a network. The communication and measurement bandwidth of the whole localization network system is 150 Hz, as defined in the PANS protocol. This means that 750 tags can be located at a rate of 0.2 Hz sampling frequency, 150 tags at 1 Hz, or 15 tags at 10 Hz (maximum sampling frequency). The minimum sampling frequency is one minute, which would theoretically allow for work with 9000 tags. The maximum number of visible anchors is 30. This does not mean that there can only be 30 anchors in a network. It is possible to include more anchors if they are far enough away and outside the UWB range. The restriction is related to the fact that each *master* anchor can only see a maximum of 29 regular anchors.

Listeners are used to gain access from a central point to a complete view of all tags within a network. A listener is a tag with the UWB radio in passive mode (with the UWB link used for communication but not for range measurement), and connected to a PC via USB (green node in Figure 2b). A limitation of this access mode is that it can be used to see the positions of all tags but not their respective measured ranges.

In addition to this, gateways can be used to extend the network by deploying anchors over longer distances (with no upper limitation) in order to cover larger buildings (Figure 2c). Gateways are normal nodes connected by IP (Internet Protocol) to a PC. This is done by attaching a motherboard of a DWM1001DEV (configured as a *bridge*) to a Raspberry PI3 using the connector available for this purpose. One hardware limitation set up by the manufacturer is that the master node can not see more than 30 anchors simultaneously. As stated above, gateways can only see the position (but not the ranges) of all tags, but not their individual measured ranges.

### 2.2. The Pans Protocol Implemented in Dwm1001 for Range Measurement

The PANS protocol (Positioning and Networking Stack) by Decawave is a software that defines how the interactions within a DWM1001 node network are orchestrated in order to measure ranges between nodes and communicate information over the UWB channel.

**Figure 3:**
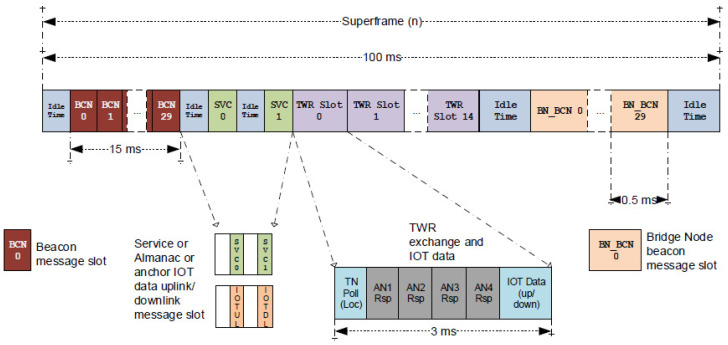
Superframe of Decawave’s PANS protocol for its DWM1001 modules. The protocol limits to four anchors the maximum number of ranges that each tag can measure within its TWR slot. However, it is quite scalable in the number of tags which can be operative at the same time—for example, 15 tags every 100 ms, or 150 tags at 1 Hz [22].

A location network based on PANS and DWM1001 modules uses time division multiple access (TDMA). At each instant of time, there is only one *tag-anchor* pair exchanging messages to measure the round-trip time (Two-Way-Ranging). Nodes operate using a 100 ms long “superframe”. This structure is shown in Figure 3. This TDMA synchronization method is very good to avoid collision, such as happens in ALOHA approaches, and guarantees a predictable scalability of the system to accommodate hundreds of tags [23].

One of the anchors is configured with the role of “initiator” (master) and is the one that keeps the clocks of all the anchors synchronized, and makes the time-slot assignments. The 15 TWR (Two-Way-Ranging) slots are used to measure the anchor-to-tag ranges and to up/download IoT (Internet-of-Things) data which may be received from or sent to a specific tag. Each TWR slot lasts for three milliseconds, and in that interval measurement of ranges up to a maximum of 4 anchors: AN1, AN2, AN3, and AN4 (as shown in the lower gray blocks in Figure 3) are allowed. If at least 4, or 3, ranges from anchors to a particular tag are available, then the tag’s position can be privately computed by its microcontroller.

**Figure 4:**
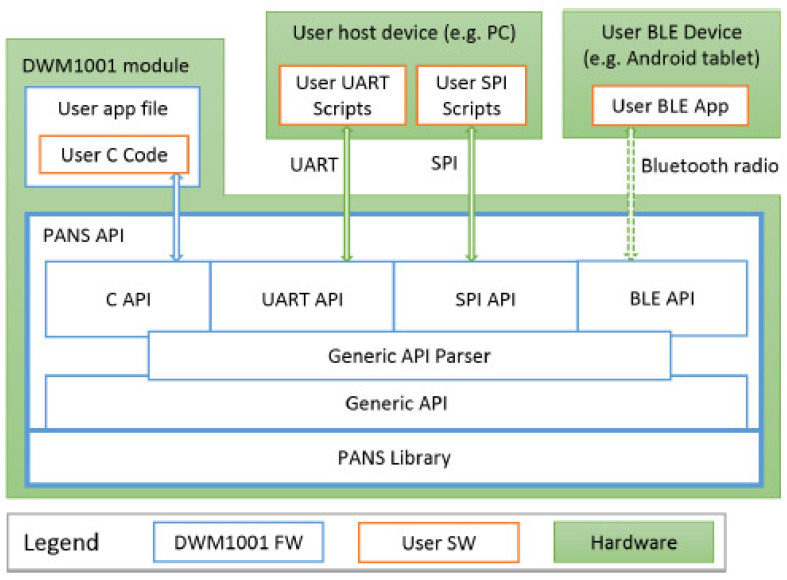
The four types of API through which we can individually access the ranges of each tag. There is an embedded API in the DWM1001 module accessible in C language, and other APIs to access by means of external equipment to the DWM1001 module through wired (serial COM) or wireless (BLE) connections [22].

At the end of the TWR (Two-Way-Ranging) slot, there are some bytes reserved for sending extra IoT information. Besides the location data, it could be possible to send or receive other data messages (range, temperature, pressure, etc.) up to a total of 34 bytes. In this way, the tag could send extra information to the network through gateways (Figure 2c). Sending IoT data such as anchor-to-tag ranges can only be achieved if the “User C Code” in each module is modified, and then making changes via the “C API” (concept further explained in next Section 2.3 and in Figure 4). On the contrary, using the listener mode (Figure 2b), you can only get access to the tag’s position, but the PANS protocol does not permit the listener to receive any data (no ranges, no temperature, …).

A set of tags and anchors constitute a network if they all have the same network identifier (PANID), the anchor being configured as “initiator” the master or coordinator. It is important to highlight that the PANS protocol allows the coexistence of several networks operating in the same space. In that case, each network will have a different identifier (PANID) and the initiating anchor will cooperate to share the time slots and not interfere with each other. Each tag will attempt to measure ranges with the closest anchors in its own network (up to four anchors), even if there is another network with closer anchors.

### 2.3. Apis for Gaining Access to UWB Ranges

The DWM1001 modules’ firmware contains a proprietary library used to create the Application Programming Interfaces (APIs) with which a user can gain access to UWB nodes. We studied the most convenient API among the four available (see Figure 4):

- *C API*. It allows a user to directly program in C language on the microprocessor existing within DWM1001 hardware, exploiting the list of functions in the documentation (“DWM1001 Firmware API guide”).
- *SPI API*. It uses a TLV (Type-Length-Value) format, which allows access to the module from an external microcontroller.
- *UART API*. Designed to communicate through a serial COM port. It is very convenient to communicate via by a USB cable a tag with e.g., a computer, tablet, or raspberry Pi. This UART API mode is simple to use. It has two operation modes: “Generic” (TLV format) and “Shell” mode that generates a terminal like prompt (dwm>) where you can send alphanumeric commands and read the system responses. To switch from “Generic” to “Shell” mode, byte 13 in decimal (CR-Carriage Return or 0D in hexadecimal) is sent twice. To switch from “Shell” to “Generic” mode, the command quit is executed.
- *BLE API*. It is the wireless mode using Bluetooth 4.0 (BLE) that allows for discovering the UWB modules that make periodic BLE broadcasts. After discovery, we can connect to them to check the offered services (standard and custom) and to read/write in their characteristics (according to protocol “Generic Attribute”-GATT of BLE). Subscription to characteristics is the most convenient way to receive a stream of ranging data from each module (tag).

**Figure 5:**
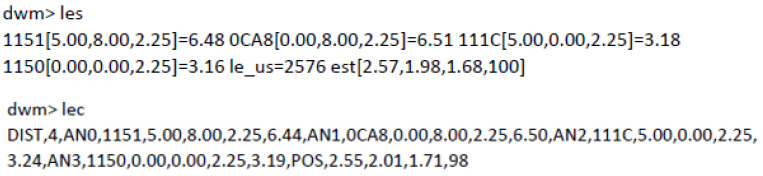
Response in the UART API (Shell) to a command les (**up**) and lec (**down**). The latter has the advantage of having a format separated by commas, which is easier to interpret.

In any of the four communication modes provided by the respective APIs, it is possible to do operations like changing the role of the nodes, e.g., configuring them as tags or anchors, activating or deactivating parameters, reading accelerometers in the tag, or reading the position. Ranges within a tag are accessible when connecting an external device to the tag, but not if connected to anchors. In “Shell” UART mode, by sending the command les or lec, it is possible to receive all the ranges (only four) and positions (if any available) of the corresponding tag continuously, until the same command is entered again, stopping that transmission. The format is a line of variable length with fields that include the positions of the anchors, the distance from that tag to each of a maximum of four possible anchors (Figure 5).

If it has been possible for the tag to calculate the estimated position, by using three or four ranges, then the XYZ coordinates of the tag is given (last four values in response to les or lec commands; see Figure 5). The field “les_us” means the time in microseconds to calculate the location. The last number in the responses is a kind of “quality factor”, which goes from 0 to 100, being a value close to 100 indicative of good quality and less than 50 of bad quality, possibly due to an NLOS condition. A more convenient indication of the quality of estimations would have been a 3 × 3 co-variance matrix for each of the spatial coordinates.

After the above revision of Decawave’s UWB positioning system, we have explored the different communications’ channels available for a user, and we have gained some insight in several limitations built into the system, which prevent us to directly use the off-the-shelf system for our research in localization. The limited number of available ranges per tag and the way to access them are the main limitations. In the next Section 3, we describe the strategies we have devised to overcome these limitations and expand the system capabilities.

## 3. Explored Alternatives for Accessing Multiple UWB Ranges

In this section, we will study ways to increase the number of read ranges. In particular, the existence of any configuration that make us able to read more than four ranges per tag. The achievement of eight ranges in a tag to 8 different anchors, we consider would be good enough to achieve the robustness we look for in positioning. It would be also useful to discover a method to access the information contained in all tags in a centralized way (without having to connect to each of the existing tags).

### 3.1. Limitations on Access to Information in Nodes

We can summarize that the limitations we have detected in the PANS protocol with MDEK1001 nodes are as follows:

- *The tags are the only ones that register ranges, and each tag only registers its own ranges.* Only the tags have information about their ranges, the anchors do not know their ranges to the tags, and there is also not a centralized place where all ranges are available. This contrasts with Decawave’s previous evaluation kit (TREK1000) [25], which, although limited to a maximum of four anchors and eight tags, used a different protocol and each of the anchors contained all the network’s information in a centralized way. Therefore, in the previous version, it was enough to get connected to a single anchor to be able to measure all the ranges between the nodes of a network. Up to 32 ranges in total (8 tags × 4 anchors) were available in the previous evaluation kit (TREK1000) but not any more in the new PANS-based MDEK1001 version.
- *Each tag measures a maximum of four ranges*. The ranges for each tag are limited to a maximum of four anchors, and these anchors cannot be selected but are chosen dynamically according to the estimated position of the tag. Another limitation is that if the tags are not able to estimate their position at a given time (due to lower than three ranges available, or inconsistency among them), the measured ranges are made with the closest anchors to position (0, 0), and not to the closest anchors to the actual location of the tag, which is a clear disadvantage.
- *There is a centralized access to all tag positions, but not ranges*. There is no centralized place where the ranges of all anchors-to-tags are received. That is, if we want to receive all the range information, we must ask each of the tags. The solutions using a listener and gateways (Figure 2b,c) designed to receive a global vision of the network state are restricted to all tags’ positions calculated by the location engine of each tag, but there is no centralized access to all ranges.

The official solution, given by the Decawave’s support to receive the four ranges of all the tags in a centralized site, is to modify the software of the nodes to include the range in the 64 available bytes of IoT data. This way, the Gateway would be able to receive those data and finally transfer them to a monitoring computer. We have discarded this solution as it requires quite a lot of development effort, and modifying the firmware of all DWM1001 nodes, the gateway and the broker/App running on the supervisor computer.

The other brute force solution to centralized ranging consists of placing an external device to each tag and picking up individually from each tag the four ranges. This solution is possible by connecting a USB cable to each tag and performing communication using UART API (Figure 5). In fact, we tried this approach, although the amount of wiring required makes this solution impractical in ordinary conditions. However, if we implement the same solution but using the BLE API by making multiple connections from a mobile, or computer with a BLE dongle, to each tag in range, it would be possible to receive the four ranges of all tags in view (at least when BLE signal quality is good). This was our final way of implementing it.

### 3.2. Design of Innovative Solutions to Measure More Than Four Ranges

As we have seen, it is not possible to read more than four ranges per tag, and there is no solution to this problem, unless someone wants to completely redesign a new PANS protocol from scratch. We have studied some ingenious ways to be able to read multiple ranges per tag in order to make a robust trilateration with redundant ranges possible. These have been our approaches to reading more than four ranges per tag:

- *Use the auto-positioning mode*. There is a self-positioning mode of the anchors where the system performs multiple mesh measurements to find out the position of the anchors. This mode can be launched from API C (dwm_loc_get command) and from the BLE API. However, the protocol performs an average of 50 measurements per range to an anchor, which implies 5 s per anchor (at the maximum 10 Hz rate), and therefore being such a slow solution we discarded it.
- *Disable anchors at intervals*. If we could disable certain anchors in the network iteratively to force the tag to measure ranges not always with the same four anchors, and to create a variety of ranges to different anchors, we would have the equivalent of a more diversified set of ranges, albeit undersampled. This can be done by BLE changing the configuration of the anchors from “active” to “passive”, but the process takes about 2 s to be done, so the measurement frequency would be much lower, and this possibility has been discarded.
- *Duplicate anchor networks and tags*. Another simple solution, but somewhat more ingenious to make tags to operate with eight ranges, is to use two tags together, in a block, as if it were a single tag. The problem is that we duplicate the amount of tags used (double the cost), and that these tags would be connected to the same anchors of the network, so we would not obtain any advantage (only measuring twice the same thing). To force eight different ranges, we propose to create two networks with different PANIDs, but physically interleaved. This way, we have two tags together (from different networks) measuring eight ranges (each tag at four different anchors from two different networks). Therefore, this solution, which consists of creating two networks and duplicating the tags, made sense to us, and it is the one chosen to be implemented in our experimentation.

## 4. Pairing Procedure and Ranging Experimentation

To effectively pair two tags, we put them physically near. Ideally, we would put them as close as possible so they form a compact and wearable unit. However, doing so in theory could alter the radiation pattern of UWB signals, due to the mutual influence between the antennae of each tag.

**Figure 6:**
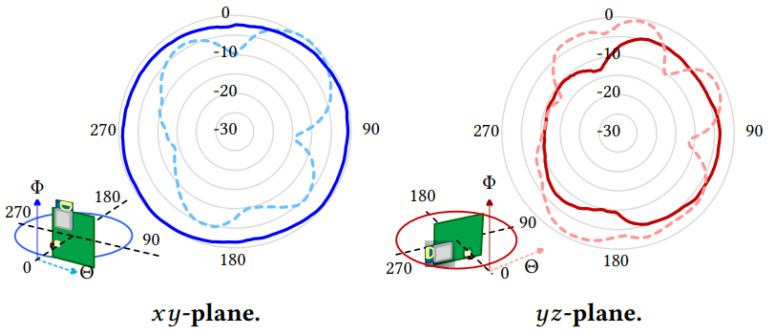
Radiation pattern of DWM1001 antennae in the horizontal (*x*-*y*) and vertical (*y*-*z*) planes with respect to the long axis of the tag’s enclosure [22,26].

The radiation pattern of Decawave’s DWM1001 antenna is omnidirectional in the horizontal plane (*x*-*y*), if both tag and anchor are oriented vertically [26]. However, the radiation pattern in the *y*-*z* plane has notches due to their design and to the on board electronic circuits (see Figure 6). According to the manufacturer [22], any metallic component closer than 10 mm to the antenna’s profile can affect the radiation pattern. Thus, when pairing the tags, we observed that separation rule.

**Figure 7:**
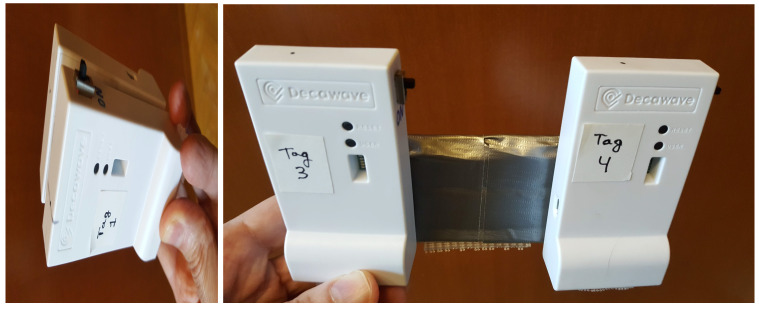
Pairing of tags: Close pairing (**left**) and Far-pairing (**right**).

In order to see how pairing between two tags could be done, we have tested two different pairing configurations, which we have called “Close pairing” (antennae separated by one inch), and “Far pairing” (antennae separated by four inches). Figure 7 illustrates these pairing choices.

The more compact “Close pairing” arrangement is very convenient and satisfies the minimum separation rule mentioned above. This is explained because the antennae in Decawave’s boxes are not placed centered between the lateral sides of the tag, but rather at the top-left area when looking at the tag from the front (just behind the letter “D” in the engraved “Decawave” name, as shown in Figure 1). Orienting the tags opposite from each other, as shown in Figure 7 (left), the antennae do not overlap, although a small sector (approximately 20 degrees wide) of the emitting pattern is shadowed by the other antenna of the pair.

The other “Far pairing” approach in Figure 7 (right) should not suffer from disturbances, since the tags are separated from each other by four inches.

In order to check these hypotheses, we performed several tests measuring the range from the pair of tags to a fixed anchor node at three different distances (1, 2, and 3 m), under LOS conditions. The two pairings were simultaneously tested on a turn table that rotated two turns (720 degrees) at each distance, while we registered the measured ranges to the fixed anchor. For comparison, we also measured ranges using a single tag on the turn table.

The *z* vertical rotation on the turn table is aligned along the antenna of one of the tags in each pair. The other tag in the pair is operative and measuring, but its data are not used for the evaluation of the propagation effects. Tags are aligned with an accuracy of a few millimeters, so any oscillation seen in the tests along the rotation are not due to misalignments in the rotation process, but just to the propagation effect of the neighboring tag in a pair.

**Figure 8:**
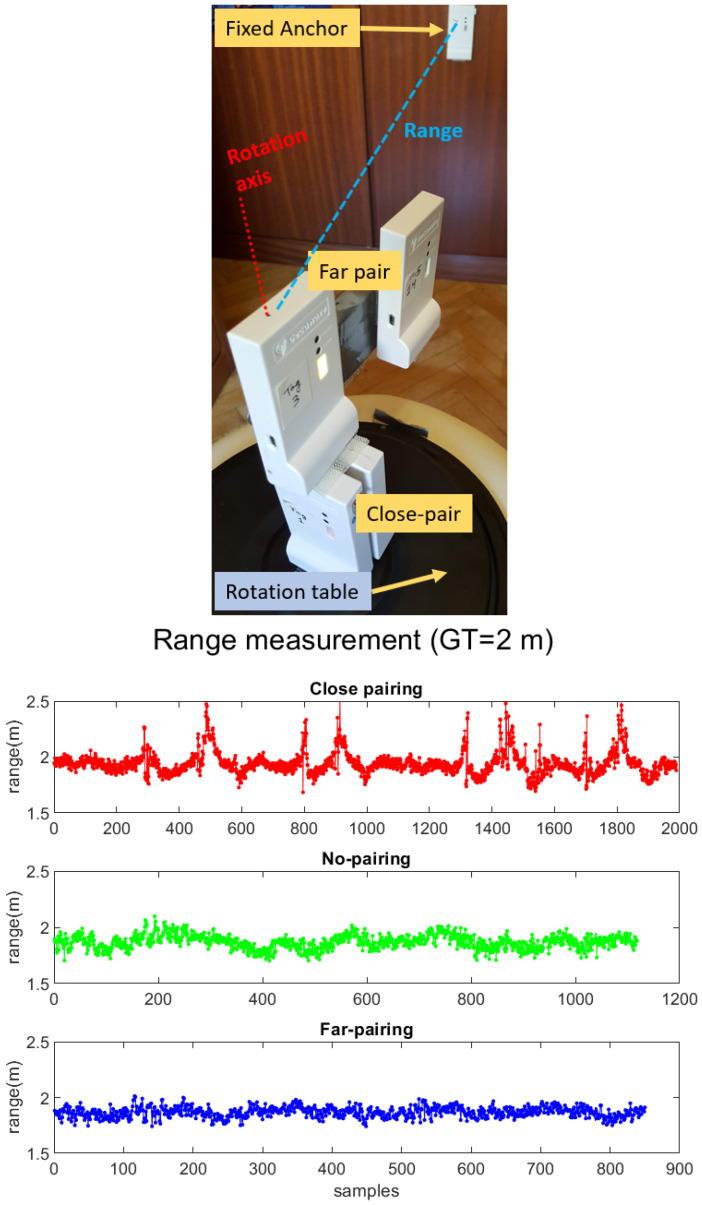

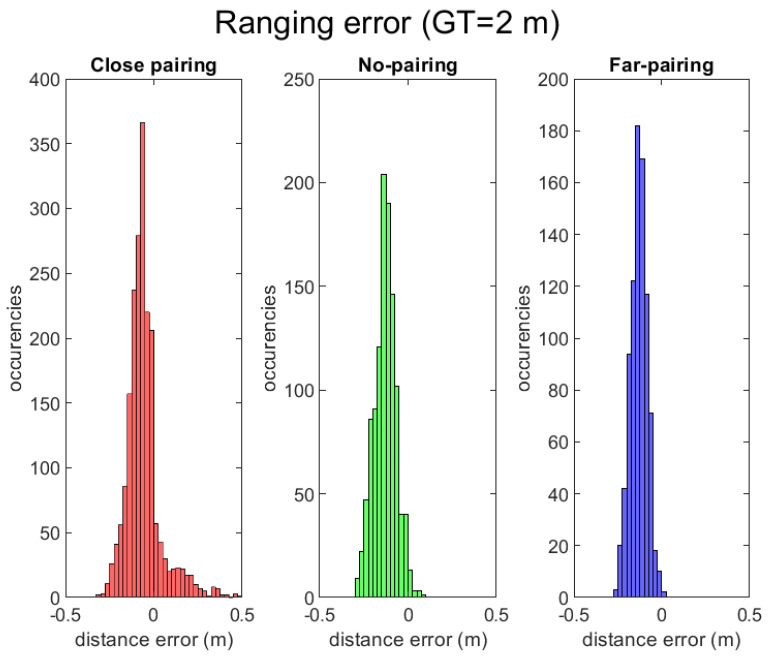
Ranging measurements at 2 m distance from a fixed anchor in LOS conditions. Effect of pairing tags in range errors for: Close pairing (red), No-pairing (green) and Far-pairing (blue).

The ranging results shown in Figure 8 suggest that the “Far-pairing” configuration performs in practice as well as a No-pairing solution (one tag alone). We found a range measurement standard deviation of 0.2 m with some bias (blue and red histograms, respectively). However, the “close-pairing” case shows occasionally in-excess range measurements of up-to 0.5 m. It can be observed in the histogram for ranging errors the tail to the right for about 15% of measurements. This range error occurs at specific rotation angles (see the regular pattern of red spikes in the range measurement plot). This confirms that the “Close-pairing” solution is good in 85% of cases, but in the remaining cases doubles the errors because of the occlusion from the neighboring pairing tag.

Although a range disturbance in 15% of the cases is relevant, we still think that “Close-pairing” is a valid configuration for location since the measurement errors do not look too disturbing when compared with the real-life obstacles that UWB signals suffer (reflection, diffraction, etc.) in indoor natural conditions. Thus, we will use both pairing methods for the localization tests presented in the next sections.

## 5. Laboratory Experimentation, Implementation of the EKF-Based Localization and Performance Evaluation

We next describe the proposed laboratory location tests designed to validate the hypothesis; then, using only four ranges is a limiting factor in indoor environments, and a redundant 8-range solution is a more powerful method to reject typical outliers in indoor environments.

### 5.1. Test Site, Equipment, and Data Capture

**Figure 9:**
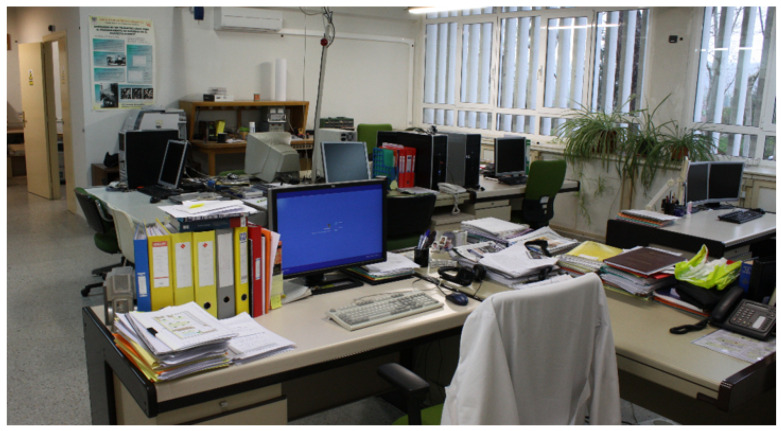
Test environment photo. Laboratory with dimensions of 11.5 m × 6.5 m. It includes a separate room with metallic furniture.

The selected test environment is our research laboratory (Figure 9), which has dimensions of 11.5 × 6.5 m (74 square meters), with diverse office furniture, and a separate room with a metallic table and cabinet. Due to these common obstacles (furniture and partitioning walls), a fraction of the round-trip range measurements between some tag positions and some anchors become NLOS (Non-Line-of-Sight). Thus, even for a relatively simple space like our lab, it includes some conditions that can cause reflection, diffraction, and attenuation on signals, generating range outliers that do not follow a Gaussian error distribution.

We have used a personal computer (PC) with Windows 10 as operating system for data capture and analysis. This computer includes a USB BLE 4.0 adapter (iAmotus BCM20702 Broadcom Inc, San Jose, CA, USA), and Matlab release 2019b from Mathworks^®^, which is the first release that includes BLE libraries to discover and open data subscriptions to BLE devices, such as our MDEK1001 tags. Although we were able to communicate with the tags through both UART and BLE, finally all the data capture tests have been done by BLE, since it has turned out to be a more practical and even more reliable solution than the wired communication with the UART API in Shell mode (where the data frame sent is larger, and the serial port buffer was frequently overflowed working at the maximum frequency of 10 Hz with several tags connected to a USB concentrator).

### 5.2. Experiments and Ground-Truth Creation

**Figure 10:**
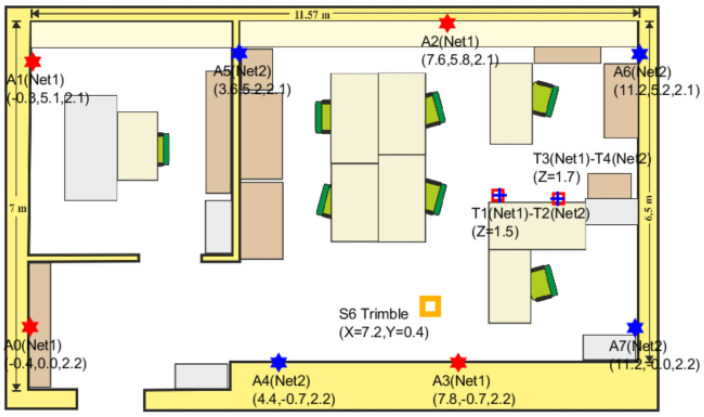
Test environment map-layout. The six-pointed stars indicate the eight anchors distributed in two interleaved sub-networks (Network1 in red, and Network2 in blue). T1-2 and T3-4 are two pairs of tags in static condition. The location of the Trimble S6 total station is marked in orange. The previous Figure 9 is a view of our lab from point A7 (bottom right corner).

For the localization experiments, we have deployed eight anchors around the lab environment (see a photo at Figure 9 for a general idea of the kind of environment, and especially look to the detailed anchor deployment in Figure 10). Anchors are stuck to the walls of the lab at a height of 2.1 m. As can be seen from the map-floor (Figure 10), four of the anchors in red (anchors A0, A1, A2, A3) are associated with the Network1, and the other four anchors in blue (anchors A4, A5, A6, A7) to the Network2. The position of each anchor has been measured using a Trimble S6 total station. This system allows us to determine the UWB antenna position in 3D with an error of one millimeter.

For a first group of tests, we installed, at fixed positions, two pairs of tags in “close-pairing” mode (being each pair able to measure up-to eight anchors). Note that each one of the tags in a pair belongs to a different network. In pair T1–T2, tag1 belongs to Network1, and tag2 to Network2. The same for pair T3–T4. The tags in a pair are stuck together (“close-pairing mode”), so, when attached to a moving person, they move as a single device.

**Figure 11:**
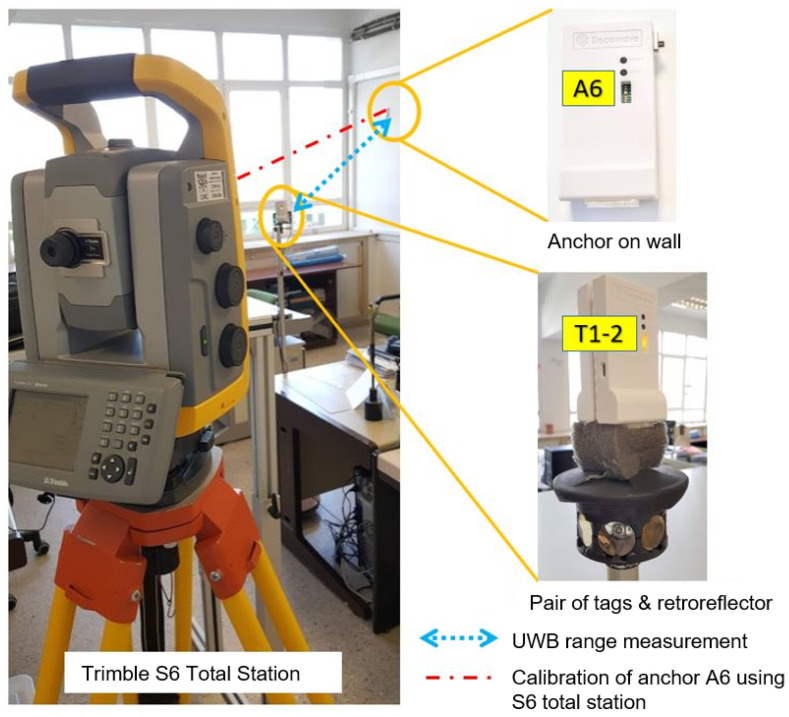
Experimental environment with the precision equipment (Trimble S6 Total Station) to calibrate the position of anchors and to track the tags obtaining the true position or GT (Ground-Truth).

For a second round of dynamic tests, the T1–T2 pair of tags has been placed on a mast, which also includes a 360° retroreflector (Figure 11 right-bottom). This setup allows us to perform free motion dynamic experiments, where the experimentation ground-truth is generated by the total station operating in *tracking mode*. This Trimble S6 tracking mode of operation performs real-time tracking by automatically commanding tilt and yaw rotations on the S6 head in order to keep tracking the retroreflector (and therefore tracking the tags on the same mast). The accuracy is 1 mm and the 3D position registration is continuous on a logfile at a 1 Hz rate. This method, which is similar to the one employed in [17], allow us to record a ground truth (GT) that is used later to make the performance location assessment. The only movement restrictions are that, during the experiment, an LOS condition should be guaranteed so the S6 head does not miss the retro and get lost; also the mast must be kept vertical to guarantee that the *x*-*y* position of the retroreflector is the same than for tags.

### 5.3. Results in Ranging Measurements

Before presenting the positioning results, in this subsection, we analyze the quality of the individually measured ranges between the different anchors and tags. We want to check the error distribution, the systematic error (bias), the standard deviation of normally distributed range errors, and the presence of outliers.

The range error has been estimated for some static tags under LOS condition and with no occlusions by human bodies. For this static condition, the error distribution was quite ideal and close to Gaussian distribution. Of more interest were the dynamic tests that provide a much broader diversity of ranging conditions, including the interaction with obstacles such as signal transmission through some brick walls or metallic cabinets.

**Figure 12:**
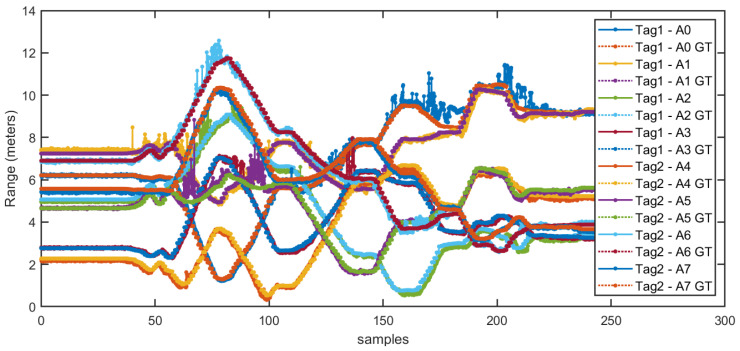
Range estimations between different anchor to tag vs. the GT (ground-truth) range.

The range estimations between different anchors to tags, for the dynamic case of a moving pair of tags within our test environment, are represented in Figure 12. We can see that, among the set of eight ranges (four anchors × 2 tags), outliers are more likely to appear when the physical range between them is larger than 6 m.

**Figure 13:**
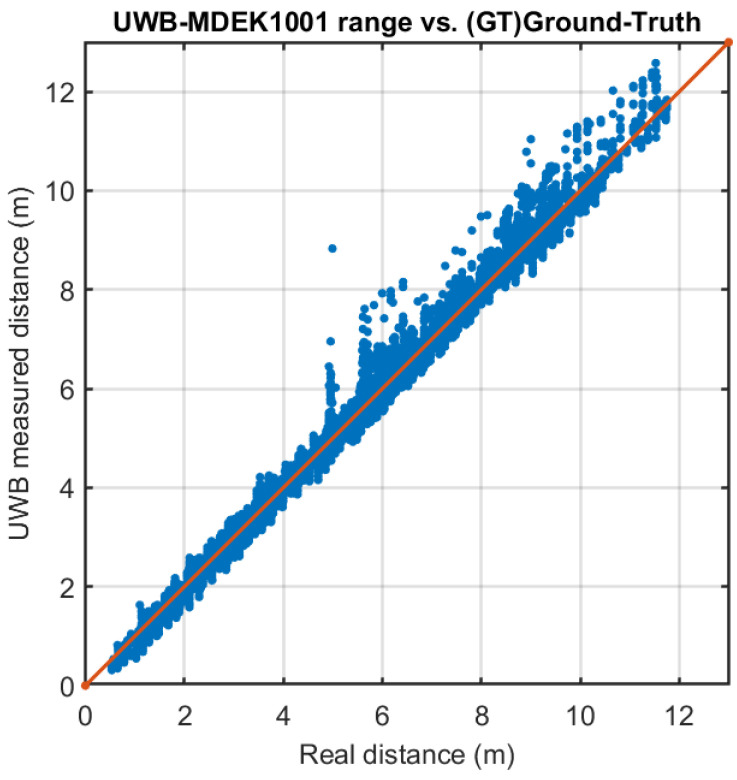

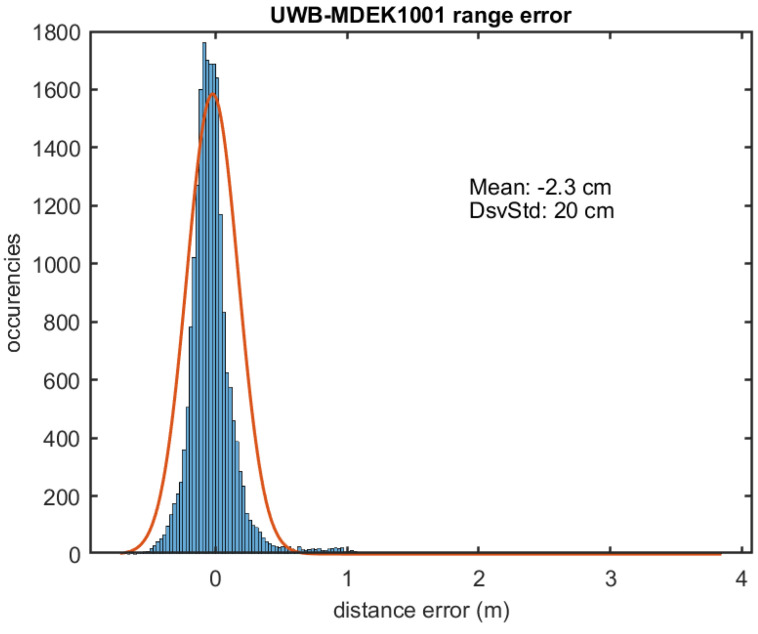
Errors in the measurement of ranges between eight anchors and four tags moving within our indoor lab space. Above: Measured vs. actual range. Below: error distribution, and overlapping Gaussian.

The range error distributions, for the same dynamic case of moving one pair of tags (as in Figure 12), can be seen in Figure 13.

The upper graph in Figure 13 shows the correlation between the real tag-to-anchor distance and the measured results using UWB ranging, with the red line with unit slope being the ideal relationship. We can see that UWB follows with quite good accuracy but with some additive noise. Figure 13 bottom shows the error distribution for the same data. The average error (bias) is −2.3 cm, i.e., the measured range is slightly below than the actual distance, and the standard deviation is about 20 cm. Some infrequent outliers appear skewed to the right in an almost imperceptible tail. Depending on the application, it might be profitable to model the tail of this distribution, or it might be better to disregard these excess ranges [11].

The detected outliers appear always in the form of in-excess ranges, as expected. These outliers have an extra range value of about 1 m, and rarely reach 4 m (worst case). Outliers are caused by NLOS conditions. Some could be caused by the presence of objects in the environment (furniture, walls, and metal cabinet), reflections, as well as by the body obstruction of the person who carries the mast. The lab conditions in our tests could be said not to be particularly adverse, as in an industrial environment, but are quite realistic with the everyday conditions in a typical office or indoor space.

### 5.4. Results in Positioning

This subsection presents the positioning assessment of the different approaches. We want to compare the positioning solution provided by the manufacturer (Decawave) using only four ranges, with our solution that makes use of up-to eight ranges by pairing two tags together. The manufacturer positioning in done locally on the tag, but the solution presented here performs a tight integration with the eight ranges by means of an Extended Kalman filter (EKF) [27].

We use a 6-state EKF, with three terms for 3D position, and three for velocity:

(1) $$X={\left(x,y,z,v_{x},v_{y},v_{z}\right)}^{T},$$

where the **motion model** is a constant velocity function *f* relating the evolution of states, plus an additive noise g(a) that takes into account the acceleration changes of the moving object:

(2) $$X=f\left(x,y,z,v_{x},v_{y},v_{z}\right)+g\left(a\right)$$

The full motion model in discrete time (dt being the sampling interval) is:

(3) $$\begin{array}{ccc} X^{k+1}= & \left(\begin{array}{c} x^{k+1}\\ y^{k+1}\\ z^{k+1}\\ v_{x}^{k+1}\\ v_{y}^{k+1}\\ v_{z}^{k+1} \end{array}\right)= & \left(\begin{array}{c} x^{k}+v_{x}^{k}dt+\frac{a^{k}dt^{2}}{2}\\ y^{k}+v_{y}^{k}dt+\frac{a^{k}dt^{2}}{2}\\ z^{k}+v_{z}^{k}dt+\frac{a^{k}dt^{2}}{2}\\ v_{x}^{k}+a^{k}dt\\ v_{y}^{k}+a^{k}dt\\ v_{z}^{k}+a^{k}dt \end{array}\right) \end{array}$$

where the state transition matrix used in the EKF is

(4) $$F=\left(\begin{array}{cccccc} 1 & 0 & 0 & dt & 0 & 0\\ 0 & 1 & 0 & 0 & dt & 0\\ 0 & 0 & 1 & 0 & 0 & dt\\ 0 & 0 & 0 & 1 & 0 & 0\\ 0 & 0 & 0 & 0 & 1 & 0\\ 0 & 0 & 0 & 0 & 0 & 1 \end{array}\right)$$

and the process noise is $G_{a}={\left[\frac{dt^{2}}{2},\frac{dt^{2}}{2},\frac{dt^{2}}{2},dt,dt,dt\right]}^{T}$.

Therefore, the process noise covariance matrix to use in the EKF is:

(5) $$\begin{array}{ccc} Q= & G_{a}q_{a}G_{a}^{T}=\; & \left(\begin{array}{cccccc} \frac{dt^{4}}{4} & 0 & 0 & \frac{dt^{3}}{2} & 0 & 0\\ 0 & \frac{dt^{4}}{4} & 0 & 0 & \frac{dt^{3}}{2} & 0\\ 0 & 0 & \frac{dt^{4}}{4} & 0 & 0 & \frac{dt^{3}}{2}\\ \frac{dt^{3}}{2} & 0 & 0 & dt^{2} & 0 & 0\\ 0 & \frac{dt^{3}}{2} & 0 & 0 & dt^{2} & 0\\ 0 & 0 & \frac{dt^{3}}{2} & 0 & 0 & dt^{2} \end{array}\right)q_{a} \end{array}$$

where $q_{a}$ is the noise in acceleration that accounts for the random changes in acceleration when the moving object changes directions and speeds. In our implementation $q_{a}=0.1\;m/s^{2}$.

For the **measurement model** in the EKF, we use the UWB ranges that we record from our BLE communication with each of the tags in a pair (eight ranges in total, four for each of the two tags). The trilateration is computed implicitly in the Kalman filter by accordingly defining measurement matrix *H*, which is of size $N_{a}\times 6$, with $N_{a}$ being the number of anchors connected to the tag at current time ($N_{a}\leq 8$). Row *i* of matrix *H* has the form:

(6) $$H_{i}=\left(\begin{array}{cccccc} \frac{x-x^{i}}{r^{i}} & \frac{y-y^{i}}{r^{i}} & \frac{x-x^{i}}{r^{i}} & 0 & 0 & 0 \end{array}\right),$$

where $r^{i}$ is the range between anchor $A^{i}$ and the last estimated tag’s position, i.e.,: $r^{i}=\sqrt{{\left(x-x^{i}\right)}^{2}+{\left(y-y^{i}\right)}^{2}+{\left(z-z^{i}\right)}^{2}}$. We use for the diagonal measurement covariance *R* matrix the experimental standard deviation detected in the last subsection (σ=0.2 m) squared ($\sigma^{2}=0.04\;m^{2}$). The trilateration process can be performed incrementally (one time for every new measurement from an anchor is received), or wait until ranges are collected for all detected anchors at a given time interval.

In order to remove most of the range outliers, and making use of the redundancy provided by the up-to eight ranges, we robustified the Kalman filter using the innovation [28]. When computing the innovation, a measurement is taken into account whenever the innovation is lower than three times the standard deviation. Thus, innovations larger than 0.6 m are not integrated, assuming that they represent outlier measurements.

**Figure 14:**
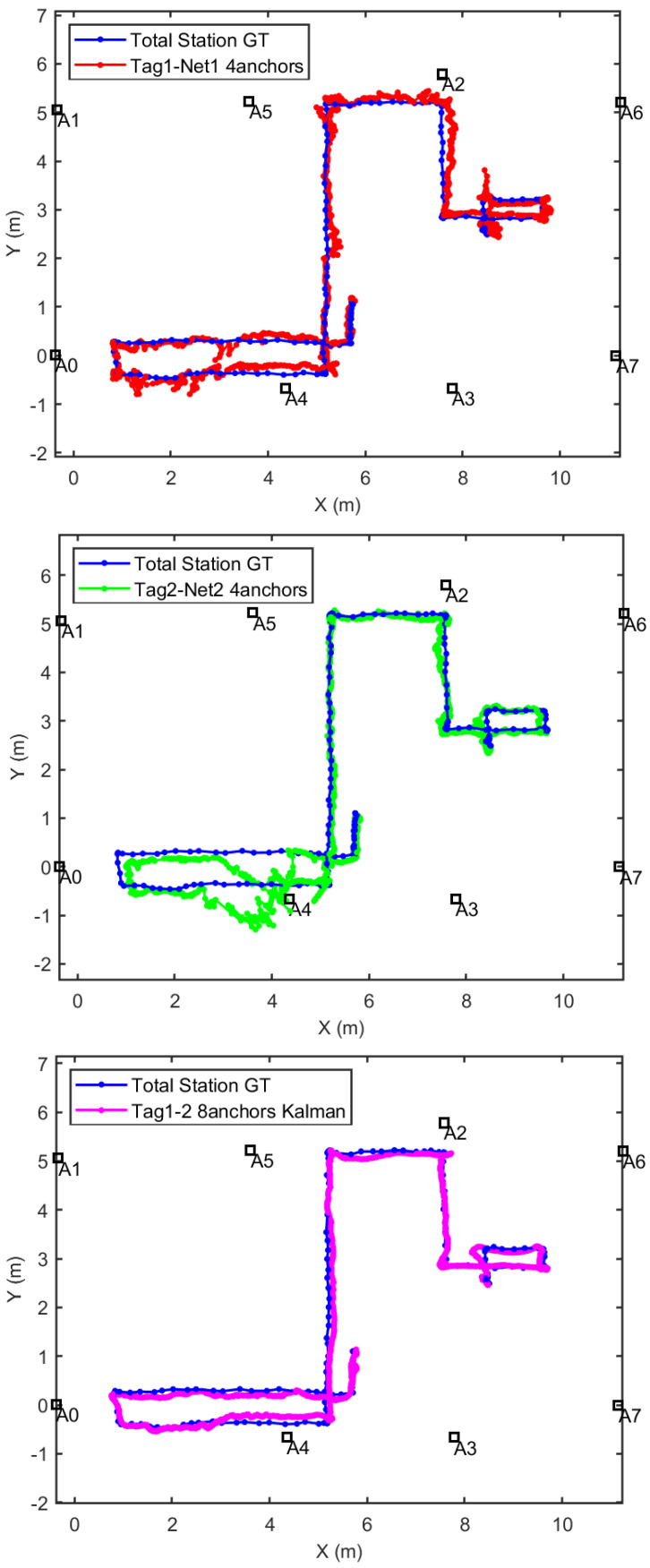
Location results with the estimation methods proposed in this work, and comparison with the ground-truth (GT) supplied by the S6 Total station. Top: Tag1 estimation by Decawave algorithm with four ranges in Network1. Middle: Tag2 estimation by the Decawave algorithm with four ranges in Network2. Bottom: Tag1-Tag2 pair estimation by EKF algorithm with eight ranges using both networks.

The positioning results for the different positioning methods studied in this paper are shown in Figure 14. The position estimation obtained with the above described EKF is called in that figure: “Kalman Tag1-2 8anchors”. On the other hand, the on-tag locally computed Decawave’s positions using only four ranges is called: “Tag1-Red1 four anchors”, and “Tag2-Red2 four anchors” for each of the subnetworks. In Figure 14, we also represent the estimates made by the Trimble S6 total station, which is considered our ground-truth (GT).

Note in Figure 14 that the estimation in magenta color (“Kalman Tag1-2 8 anchors”) is smoother than any of the four anchors’ solutions. In addition, it is important to mention that a simple averaging of both independent 4-anchor solutions does not provide as good results as our EKF tight trilateration. See, for example, the trajectory section close to anchor A4 (coordinates *x*-*y* from 4.2-0 to 2.5-0 ), where the average of the red and green trajectories does not produce the same results than the magenta one. It is clear in this example that the outlier rejection mechanism (robustification of EKF) has been able to remove the measurements with non-Gaussian errors.

**Figure 15:**
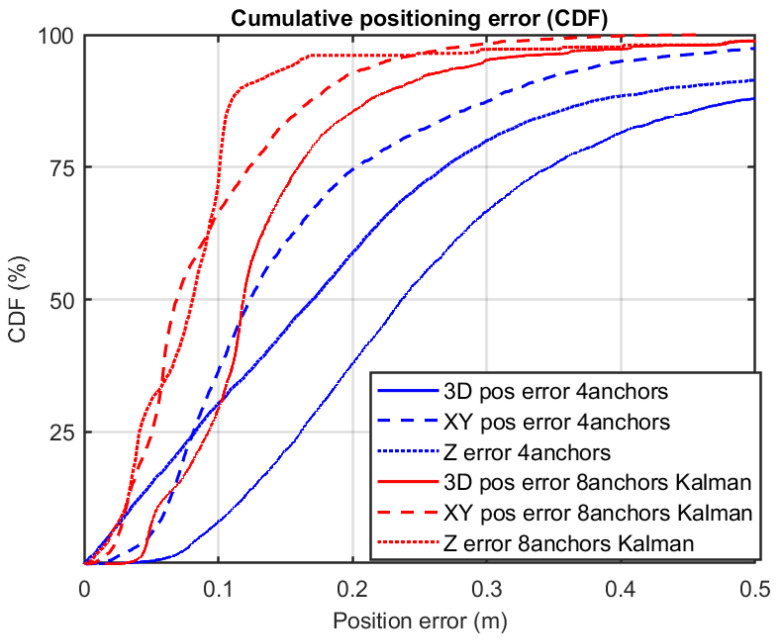
Location error in CDF (Cumulative Distribution Function), in the case of using four ranges (algorithm integrated in the tags by the manufacturer Decawave), and in the case of using eight ranges with an external algorithm based on the Kalman filter.

The detailed positioning errors are shown in Figure 15 by means of a cumulative distribution function (CDF). We can appreciate how the method proposed by the authors that integrates eight ranges is better than any of the estimations made locally at each tag with only four ranges (Decawave’s own algorithm). The 3D errors at the 75th percentile level are 16 cm and 34 cm, for the method with eight ranges and four ranges, respectively. The corresponding errors in the *x*-*y* horizontal plane are 12 cm and 20 cm, respectively. The improvement using the redundant robust estimation is about 50%, which is by far better than other approaches found in the literature that makes use of median or moving averages as in [13] that account for improvements in the range from 10 to 20%. Our positioning errors are also better than other UWB solutions using a limited number of UWB anchors but complemented with INS+GNSS, as in [17], where authors declare a horizontal error of 35 cm. Our results are closer to the performance reach using sophisticated machine learning (ML) algorithms like in [14], with errors of 0.1 m, but these results are only valid after a learning phase which depends on the particular scenario, and could not be consistent with a different or changing scenario.

It is fair to mention that the error reduction presented in this work is due not only to the increase in the number available anchor range measurements (redundancy and robustification) but also to the more favorable geometric arrangement between the location of the tag and the set of anchors (i.e., lower dilution of precision or DOP [29]).

At this point, we could conclude that a location solution using more than the four ranges allowed by PANS protocol is preferable for indoor scenarios. However, this experimentation was done in a relatively ideal scenario and for a limited number of trajectories. In order to obtain more solid conclusions, we could perform more trajectories in the same laboratory or close-by spaces, or we could even change the position of anchors. However, we consider it much more interesting to create a new deployment in a totally different sites, with a much higher content of obstacles causing NLOS effects and many range outliers. The next section describes this experimentation.

## 6. Apartment Experimentation

In order to check if the performance obtained in the laboratory test site is valid in other more challenging scenarios, we have made additional tests at a totally different site: a residential apartment. The next subsections review the scenario details, the ranging, and the localization results, and provides a comparison with the previous laboratory tests.

### 6.1. Apartment Test Methodology and Sensor Deployment

This new environment is an 80-square meter apartment, fully furnished and with common household objects, where one of the co-authors lives. We made an initial eight anchors deployment, placing anchors at 2.14 m height (mainly on top of the door frames), but, due to the diverse number of brick walls, wardrobes, electric appliances, and ornaments, the UWB coverage was not complete. We added four new anchors, for a total of 12, and we reached the minimum desired coverage for both the 4-range commercial solution and the extended up-to-eight ranges with EKF localization.

**Figure 16:**
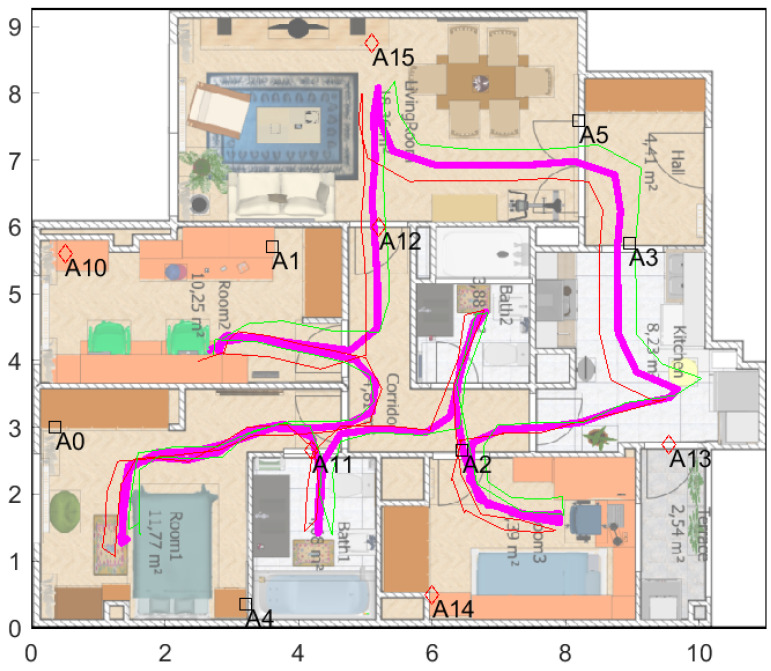
Apartment’s floormap. A total of 12 anchors were deployed creating two networks of six anchors each (Net1: squares, and Net2: diamonds). A ground-truth for one of the experiments is marked with a magenta line.

Figure 16 shows the apartment’s floormap with the 12 deployed anchors. Six of the anchors are annotated with a square marker (anchors A0, A1, A2, A3, A4, and A6) because they belong to one of the duplicated networks (Network1), and the other six anchors marked with a diamond (anchors A10, A11, A12, A13, A14, and A15) belong to Network2.

The ground-truth (GT) in this occasion was not done with a Total-Station (impractical due to the lack of line-of-sight to the mobile tag from a base station). Thus, a less accurate GT was obtained with a pair of foot-mounted inertial motion units (IMU), one on each shoe, and use of the Pedestrian Dead Reckoning (PDR) technique [30]. This provides relatively accurate trajectories, with low drift for the lengths considered in the experiments. The position of each anchor, as well as the map calibration, has been done with a laser-range finder (Bosch GLM-80 Professional), both on the *x*-*y* plane and in vertical over the floor (wherever possible anchors are at a 2.14 m height).

The magenta line shown in Figure 16 represents the IMU-based GT used for one of several tests in this scenario. This reference is created by averaging the trajectories of each independent foot. As IMU-based estimation is relative (dead-reckoning), a post-processing is done in order to rotate and translate the origin of the trajectory to the starting point. In all trials, the starting and ending point was the same (Room2, near anchors A10 and A1).

The IMUs used for GT registration were model MIMU22BL from Inertial Elements, India [31]. They consist of an array of four IMUs (ICM-20948 TDK Invensense), whose sensor readings are internally processed by a 32-bit microcontroller (Atmel AT32UC3C0512C), resulting in a step-wise dead-reckoning (SWDR) data, which is given as output of the IMU. The SWDR data consists of the incremental changes in the xyz-coordinates per step detected, as well as yaw changes, at a step-like rate of approximately 1 Hz.

The MIMU22BL has two communication interfaces: Bluetooth v4.1 (BLE) and USB 2.0. The protocol used for data recording during experimentation is BLE, both for the IMUs and the UWB tags. Data are acquired by a laptop computer with Matlab R2019b (or the GetSensorData android App [32]), where corresponding objects are instantiated from MIMU22BL and MDEK1001 classes. After a sequential BLE connection, the “start” method for all objects is executed, and data logging is achieved with a common time-stamping which permits synchronization.

**Figure 17:**
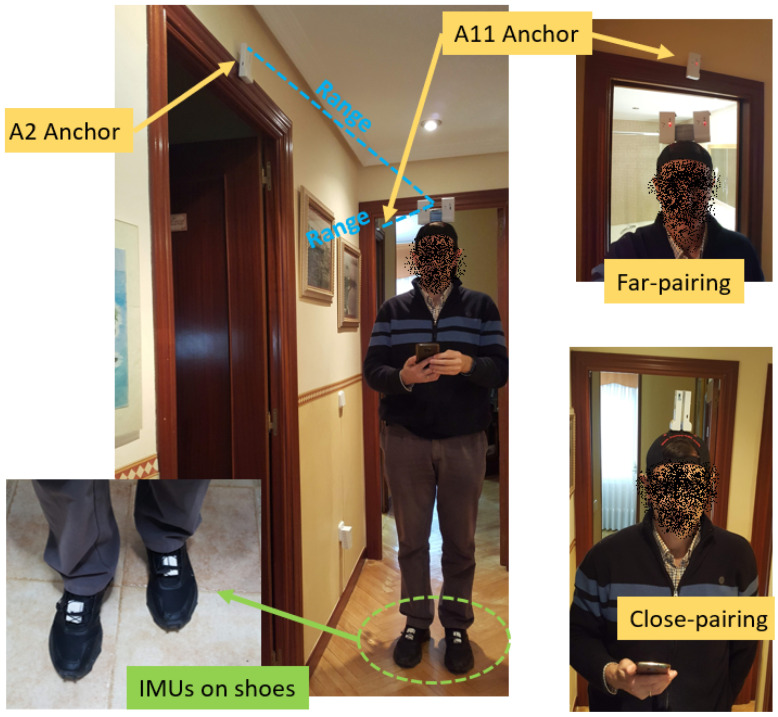
Apartment details, showing some examples of anchors locations, tags mounted on a cap on the actor’s head in both pairing configurations, and IMUs attached on shoes.

We can see the IMU placement on the shoes of the actor, and other details of UWB tag/anchors installation at the apartment in Figure 17.

The IMU-based GT trajectories are accurately synchronized with UWB readings, but are by-far not as accurate as those obtained with a Total Station in the laboratory setup. We have observed typical positioning errors of about 0.2 m, with a few excursions up-to 0.5 m in some zones. However, taking into account that the measured UWB ranges in the apartment are less precise, as will be seen next, the IMU-based GT is a good enough reference to assess the performance of the localization solution.

### 6.2. Apartment Tests’ Data Analysis

**Figure 18:**
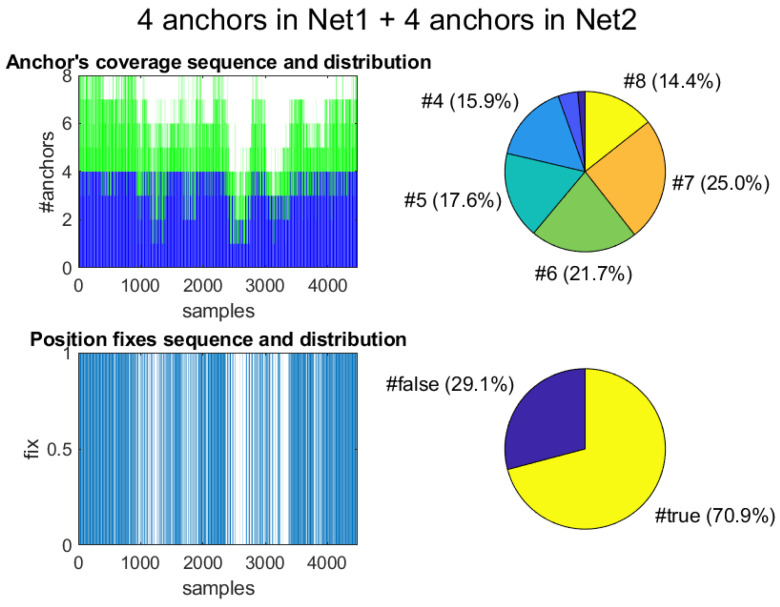
Coverage of anchors for one of the testing routes at the apartment. Top: Total number of anchors in view when considering the contribution of both tags in a pair (tag1&tag2). Bottom: Position fixes computed by the Decawave algorithm based on four anchors at a time.

We performed tests with both the far-pairing and close-pairing of tags. In both cases, we used the 12 deployed anchors to cover the complete area, but, as explained before, only eight are detectable at a given time. Thus, the maximum amount of ranges was eight (four from tag1 and another four from tags 2). The typical coverage in the apartment is illustrated in Figure 18. The top-left plot represents the number of anchors under view during a complete session (more than UWB 4000 samples at 10 Hz). Blue and green lines represent the contribution from each of the tags. We can see that the full 8-anchor coverage is almost never fulfilled. However, as we can see in the pie chart (top-right), in 14.4% of the cases, eight ranges are read. In fact, we can read more than four ranges in more than 75% of the cases, thanks to pairing.

The temporal windows between samples 1000–1500 and 2500–3500 (Figure 18 top-left) correspond to visits to rooms with a bad quality in ranging. These rooms are Room3, Room1, and Kitchen. In these areas, there is a significant drop in coverage, or anchor visibility, but it is not due to a lower density of deployed anchors, which is quite even for the whole apartment; it is just an empirical circumstance.

On the other hand, we also see in Figure 18 (bottom-left and right) that the number of position fixes provided by the commercial Decawave algorithm is not complete along the experimentation. In 29.1% of the cases, no trilateration-based position is generated by using a maximum of four ranges per tag. Position fixes fail when two or less ranges are available but also when three or four ranges are present, but the quality of the solution is low (Decawave provides a fix quality indicator). However, the EKF method always provides 100% position fixes, since, by design of the filter, a position estimate can be produced even in the absence of range information, based on previous data.

**Figure 19:**
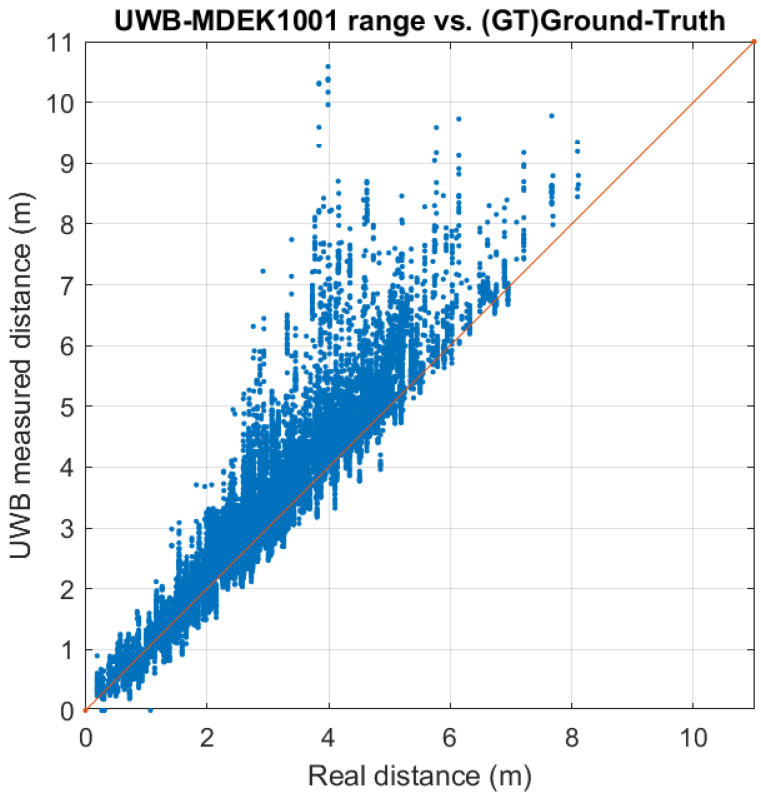
Real-distance vs. UWB measured distance (tag to anchors) at the apartment scenario.

When we look at the ranging errors, we expect to have larger errors than in the laboratory tests, and also a lower maximum read range, due to the abundance of walls and obstacles that make UWB signal propagation difficult. This is exactly what we can see in Figure 19, where very few measurements are registered beyond 7 m. If we look back at the same plot in Figure 13, we see that in a site of approximately the same size (74 square meters) ranging was effective up-to 12 m. Thus, the apartment site looks more challenging.

**Figure 20:**
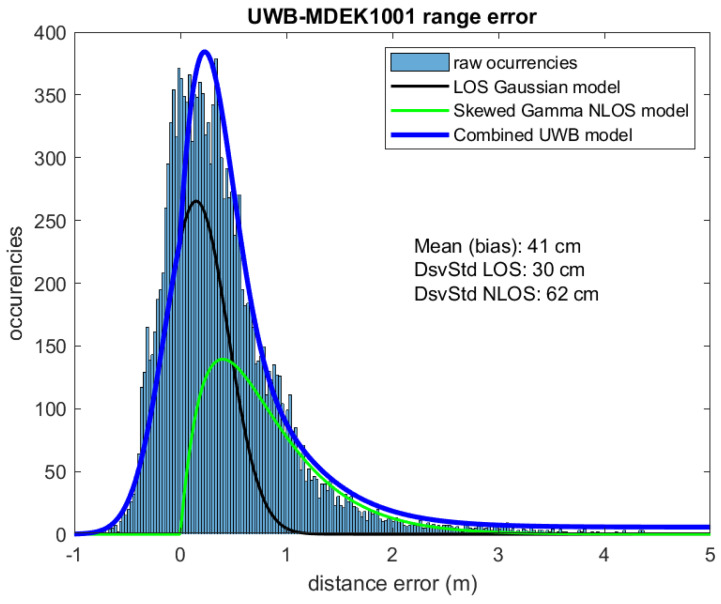
Error distribution in ranging (tags to anchors) at the apartment scenario An NLOS model (blue) is overlaid on the experimental histogram.

Plotting the error distribution will show an asymmetrical peak near zero, followed by a long skewed tail, as seen in Figure 20. This situation is typical of mixed LOS / NLOS situations such as those found in the apartment site. The resulting error probability distribution function (PDF) J(x) for positioning error *x* is modeled as a sum of two contributions [33]:

(7) $$J\left(x\right)=\frac{1}{\sigma \sqrt{2\pi }}e^{-\frac{{\left(x-\mu \right)}^{2}}{2\sigma^{2}}}+\lambda \cdot e^{-\lambda x}\cdot \frac{{\left(\lambda x\right)}^{k-1}}{\Gamma (k)}+cte,$$

where the first term corresponds to LOS situations, with an assumed Gaussian distribution, and the second to NLOS situations, usually modeled by a Gamma distribution. The last, constant term (cte) represents the additional uncertainty and spurious measurements.

We performed a least-squares fit for the free parameters of Equation (7) using the experimental data. For the LOS contribution, we obtained σ=0.2 m (standard deviation of range error) and μ=0.15 m (bias of the range); for the NLOS part, we obtained λ = 2.5 1/m (*rate* or inverse *scale* of a Gamma PDF) and *k* = 2 (the *shape* parameter). The last term, *cte*, was assigned a value of 1.5% of the model’s peak. The result of the fit for the combined distribution is shown overlaid on the histogram data in Figure 20.

Note that the error plot and distribution shown in Figure 19 and Figure 20 are in part caused by the location drift errors of the IMU-determined ground-truth, and not by UWB ranging only. However, the effect is not expected to be too relevant since we are observing ranging errors larger than the typical 0.2 m position drift error for the GT.

**Figure 21:**
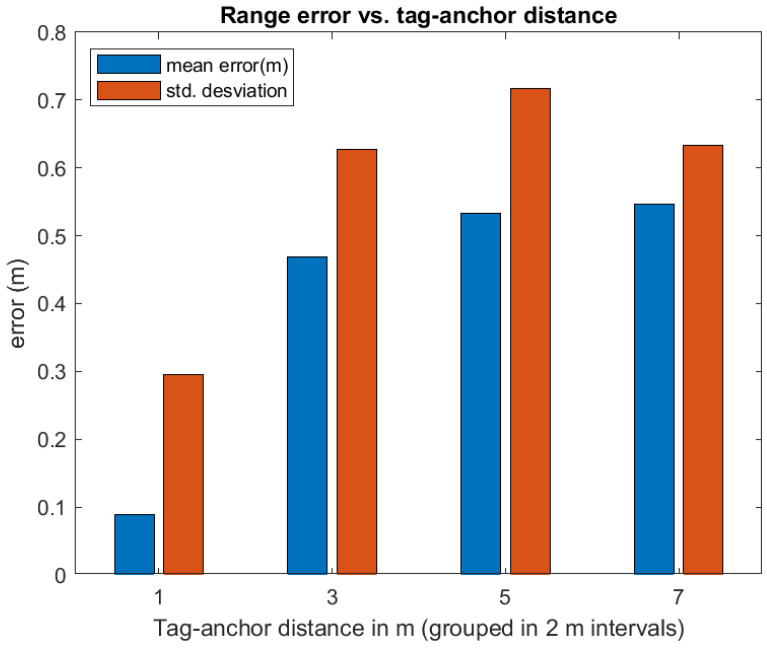
Mean and standard deviation of the measured range error in four groups of range bins: 0–2 m, 2–4 m, 4–6 m, and 6-Infinite. Information is obtained from the same data as in Figure 19 and Figure 20.

We have also studied the dependence of range error with respect to the true range. From physical reasons, we expect both error bias and variance to increase with range in the apartment, as LOS propagation becomes more likely. This effect is shown in Figure 21, where the ranges are grouped in four bins (0–2 m, 2–4 m, 4–6 m, and 6-infinite). We clearly see that, for ranges bigger than 2 m, the mean error and variability of ranges grow significantly, as the UWB signals have to penetrate one or more walls.

The progressive lower quality of range estimates with increasing range is a problem for an 8-ranging system (as the one proposed), which works with close-by anchors but also with anchors further away. From that noise-centric point of view, it could be better to work only with the closest ranges; however, we believe that despite the fact that useful information still remains in the more degraded long-range measurements. The exploitation of that degraded information should be conveniently integrated and filtered into a position fix. The localization results that come next will give us an answer to this important fact.

### 6.3. Apartment Localization Results

The Decawave’s integrated localization algorithm is performed inside the tags when a number of three or four ranges are available. In our tests with the Decawave’s trilateration algorithm, no position fix is detected in 29.1% of the cases, using the interleaved double network deployment. Under this circumstance, four ranges must be selected from a total of six anchors available in a network. The failed position-fix rate is improved to 16.2% when all 12 anchors are associated with the same network, so the tags can select four anchors from 12 anchors available. This improvement was expected, but it is surprising that still many position fixes can not be achieved when three or more ranges are available in 98% of the cases. On the contrary, our EKF solution working with up-to-eight ranges achieves a 100% coverage even when, in 2% of the cases, there were two or less ranges visible.

The EKF solution uses the same motion and measurement models that were presented in Section 5 for the laboratory tests. The standard deviation for measurements (used for the *R* covariance matrix) is 0.2 m, which is the typical UWB error distribution in LOS conditions. However, this time we have used a robustified innovation filter which removes ranges that deviate more than three times sigma (or 0.6 m) from the predicted range. This step seeks to remove those ranges in the long tail represented by the NLOS exponential measurement model (see Figure 20), and keep ranges likely to be LOS or at least not too affected by obstacles.

**Figure 22:**
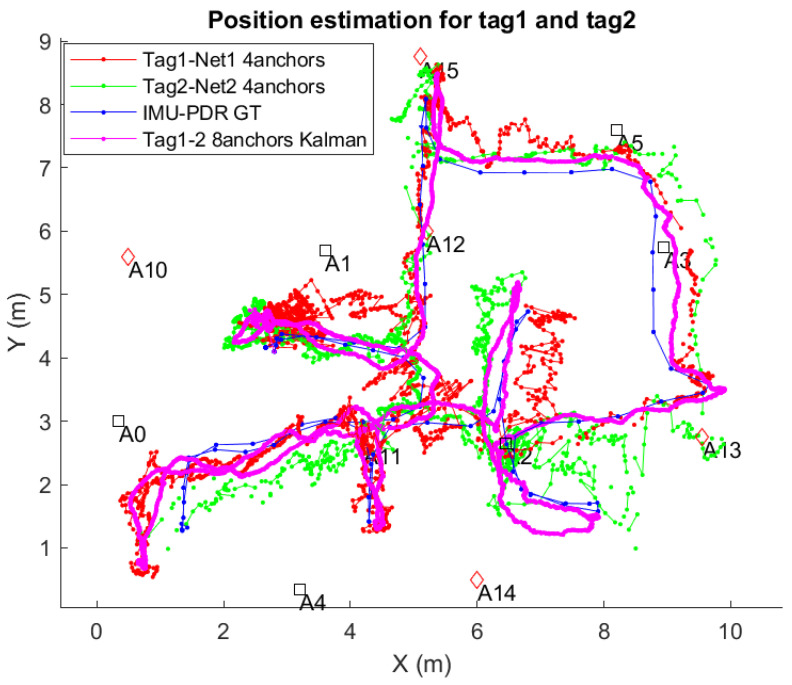
Localization results at the apartment site.

The positioning accuracy that we found from our experiments is represented in Figure 22. The location solutions presented are for the near-pairing tag mounting, but similar results are obtained in the far-pairing configuration. We do not repeat them for sake of simplicity, since the conclusions are the same.

In Figure 22, the GT is represented with a blue line, with some dots representing the steps during the IMU-based GT creation. In this plot, the red and green lines are the position fixes generated internally by Decawave’s algorithm at the two tags within a pair. The broken lines that occur in 29.1% of the cases are measurements with some ranges but with invalid position fix (not generated by Decawave’s tags).

**Figure 23:**
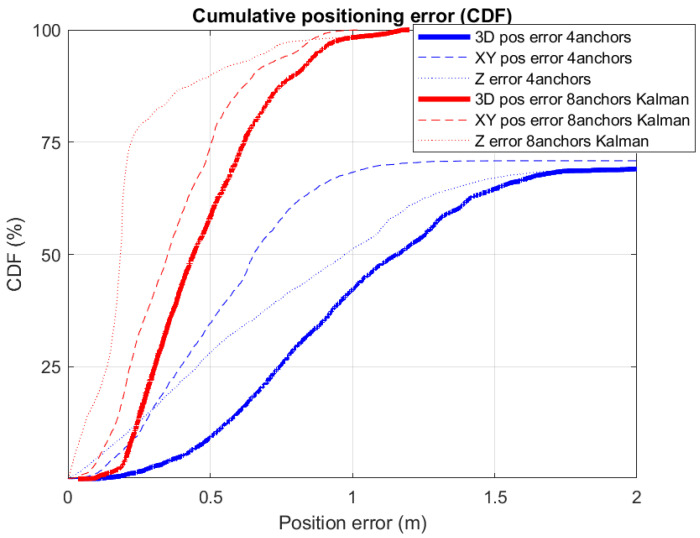
Cumulative Distribution Function (CDF) of the positioning error obtained from the localization experiment in Figure 22.

The magenta line in Figure 22 is the output from the EKF that uses all raw ranges available at both tags (up to eight ranges). Thanks to the prediction phase of the filter, we always count with 100% coverage, even when ranges to anchors are below 3 or even no range at all is present. That is not a merit of the proposed pairing process and interleaved anchor networks, but a feature of the EKF filter, as well as the smoothing effect on the trajectory provided by the filter. The important thing to see is if the use of more ranges helps in reducing positioning errors, even knowing that they can arrive from distant anchors. To analyze this, we present in Figure 23 the Cumulative Distribution Function (CDF) of positioning errors.

We can see in the CDF of Figure 23 that the 3D-positioning error for the third quartile (75% of measurements) is 0.6 m for the 8-range EKF solution (red curve), clearly better than the 4-range solution shown in blue. In this last case, the CDF does not even reach the third quartile since, as stated previously; for 29.1% of the cases, there is no position fix returned by Decawave’s algorithm. If for this setup, we only take into account the errors from the set of generated position fixes, then the third quartile error is 1.05 m. Consequently, we can observe a clear improvement in positioning from 1.05 m error to 0.6 m, obtained by adding extra ranges to the position estimate. Thus, the method proposed not only permits to use more ranges if available, but also exploits isolated ranges at locations with less measured ranges than the three required for trilateration, and where Decawave’s system does not provide a solution at all. The result is a better and more continuous localization.

One of the reasons for this improvement in our proposal is the use of redundancy. Although we all deal with ranges that have significant noise, some of them are outliers that include errors larger than 1 m, as can be seen in Figure 20. Outliers spoil the solution or make it inconsistent. The redundancy is a very important achievement that makes a better rejection of bad ranges possible. Those ranges can be included in an NLOS model and weighted accordingly (e.g., with a particle filter [25] or static/dynamic models [34]), but also can simply be deleted from our trilateration (the current approach in the robustified EKF described previously). In our experiments, about 11% of the ranges are eliminated from the Kalman filter update process (1634 rejections from a total of 15,201 range measurements). This is the key for improving the solution, and it was made possible thanks to the redundancy provided by our multiple-ranging approach.

## 7. Conclusions and Future Work

In this article, we have presented a study of some limitations of Decawave’s MDEK1001 commercial positioning system, specially originated from the PANS protocol. We have seen that PANS protocol is not prepared to work with more than four ranges for each tag to be located, which is a serious hindrance for operation of the system in real indoor scenarios. To overcome this limitation, we have proposed different ways to be able to use more ranges (eight ranges in particular). Finally, we have implemented a solution that consists of doubling the number of operating networks and the number of tags. We proposed and evaluated several configurations to pair tags. We presented the implementation of a robustified Kalman filter (EKF) to integrate multiple and redundant range measurements, based on NLOS models and an innovation-based range rejection process. Experimental tests have been carried out on UWB ranging and location performance. Two different scenarios were used, one in our laboratory, which is more favorable for UWB positioning (where conditions are mostly LOS), and another test site in an apartment, which has severe NLOS effects. The calibration of the deployments and GT trajectories were done with a Trimble S6 total station and a foot-mounted IMU on each shoe of an actor (at laboratory and apartment sites, respectively). From these new configurations (tag pairing, double anchor network, NLOS-rejection EKF) and the detailed experiments, we have been able to show that a redundant multi-range UWB solution makes the use of redundancy to create robust estimators possible and consequently improves localization performance.

This work makes fundamental and practical advances over the state of the art by offering a way to make possible the use of multiple ranges in one of the most used and successful pieces of UWB equipment in research labs and corporate solutions. An important contribution of the paper is that it demonstrates with two very different deployments how the same approach (same sensors, EKF parameters, and NLOS models) can be applied in totally different scenarios with the same rate of performance improvement. The need of learning or adaptation in the algorithms for different scenarios (common in many research papers [15,16]), is something that we were able to avoid, which is very convenient and practical in real life applications.

In the future, we will continue to explore the performance of UWB-based localization, using other robust filtering implementations, the integration with inertial measurements, and the extension to other strategies for both indoor and outdoor environments. One particular application area is in the remote supervision of the activity of people in their homes, analyzing their mobility and gait cycle (useful in heath applications such as frailty detection in the elderly). Another particular area we want to work in is the navigation of autonomous vehicles, especially in outdoor areas with poor GNSS coverage. Thanks to the good accuracy, low latency, and high measurement frequency of UWB solutions (up to 10 Hz in Decawave’s case), we envision that UWB technology could achieve similar performance to high cost GNSS/RTK (real time kinematics) systems used for accurate outdoor navigation of autonomous vehicles.

## Data Availability

Not applicable.

## Abbreviations

The following abbreviations are used in this manuscript:

API	Application Program Interface
BLE	Bluetooth Low Energy
CDF	Cumulative Distribution Function
EKF	Extended Kalman Filter
GT	Ground-Truth
IMU	Inertial Measurement Unit
LOS	Line-of-Sight
ML	Machine Learning
NLOS	Non-Line-of-Sight
PANS	Positioning and Networking Stack
UWB	Ultra-Wide-Band
